# Beyond the loss of beta cells: a quantitative analysis of islet architecture in adults with and without type 1 diabetes

**DOI:** 10.1007/s00125-025-06376-9

**Published:** 2025-02-26

**Authors:** Nicolás Verschueren van Rees, Peter Ashwin, Conor McMullan, Lars Krogvold, Knut Dahl-Jørgensen, Noel G. Morgan, Pia Leete, Kyle C. A. Wedgwood

**Affiliations:** 1 Department of Mathematics and Statistics, University of Exeter, Exeter, UK; 2 EPSRC Hub for Quantitative Modelling in Healthcare, University of Exeter, Exeter, UK; 3 Living Systems Institute, University of Exeter, Exeter, UK; 4 Exeter Centre of Excellence for Diabetes Research, Department of Clinical and Biomedical Science, University of Exeter Medical School, Exeter, UK; 5 Division of Childhood and Adolescent Medicine, Oslo University Hospital, Oslo, Norway; 6 Faculty of Medicine, University of Oslo, Oslo, Norway

**Keywords:** Human, Image analysis, Immunopathology, Islet architecture, Machine learning, Mantle–core, Pancreas, Quantitative, Shareable tool, Type 1 diabetes

## Abstract

**Aims/hypothesis:**

The organisation and cellular architecture of islets of Langerhans are critical to the physiological regulation of hormone secretion but it is debated whether human islets adhere to the characteristic mantle–core (M-C) structure seen in rodents. It is also unclear whether inherent architectural changes contribute to islet dysfunction in type 1 diabetes, aside from the loss of beta cells. Therefore, we have exploited advances in immunostaining, spatial biology and machine learning to undertake a detailed, systematic analysis of adult human islet architecture in health and type 1 diabetes, by a quantitative analysis of a dataset of >250,000 endocrine cells in >3500 islets from ten individuals.

**Methods:**

Formalin-fixed paraffin-embedded pancreatic sections (4 μm) from organ donors without diabetes and living donors with recent-onset type 1 diabetes were stained for all five islet hormones and imaged prior to analysis, which employed a novel automated pipeline using QuPath software, capable of running on a standard laptop. Whole-slide image analysis involved segmentation classifiers, cell detection and phenotyping algorithms to identify islets, specific cell types and their locations as (*x,y*)-coordinates in regions of interest. Each endocrine cell was categorised into binary variables for cell type (i.e. beta or non-beta) and position (mantle or core). A χ^2^ test for independence of these properties was performed and the OR was considered to estimate the effect size of the potential association between position and cell type. A quantification of the M-C structure at islet level was performed by computing the probability, *r*, that the observed number of non-beta cells in the mantle is due to a random arrangement. The distribution of the *r* values for the islets in the study was contrasted against the *r* values of a digital population of equivalent randomly arranged islets, termed digital siblings. Both distributions of *r* values were compared using the earth mover’s distance (EMD), a mathematical tool employed to describe differences in distribution patterns. The EMD was also used to contrast the distribution of islet size and beta cell fraction between type 1 diabetes and control islets.

**Results:**

The χ^2^ test supports the existence of a significant (*p*<0.001) relationship between cell position and type. The effect size was measured via the OR <0.8, showing that non-beta cells are more likely to be found at the mantle (and vice versa). At the islet level, the EMD between the distributions of *r* values of the observed islets and the digital siblings was emd-1d=0.10951 (0<emd-1d<1). The transport plan showed a substantial group of islets with a small *r* value, thus supporting the M-C hypothesis. The bidimensional distribution (beta cell fraction vs size) of islets showed a distance emd-2d=0.285 (0<emd-2d<2) between the control and type 1 diabetes islets. The suffixes ‘-1d’ and ‘-2d’ are used to distinguish the comparison between the distribution of one and two variables.

**Conclusions/interpretation:**

Using a novel analysis pipeline, statistical evidence supports the existence of an M-C structure in human adult islets, irrespective of type 1 diabetes status. The methods presented in the current study offer potential applications in spatial biology, islet immunopathology, transplantation and organoid research, and developmental research.

**Graphical Abstract:**

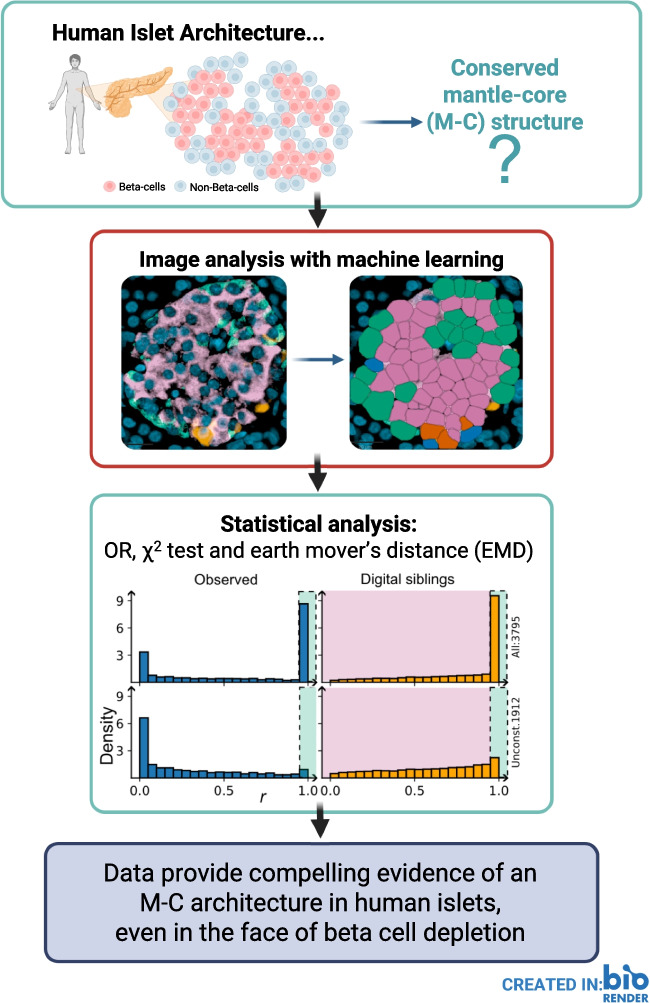

**Supplementary Information:**

The online version contains peer-reviewed but unedited supplementary material available at 10.1007/s00125-025-06376-9.


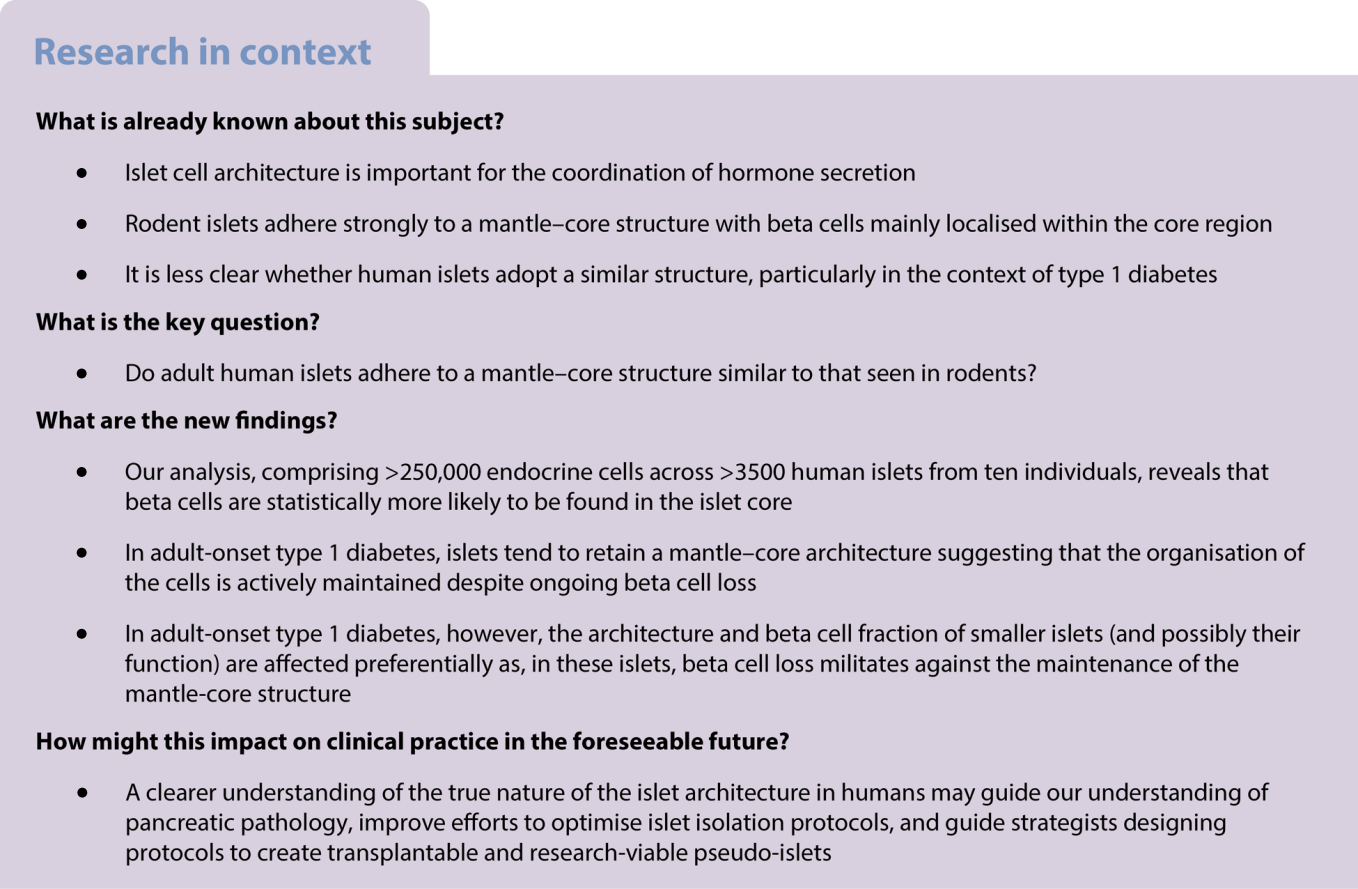


## Introduction

Type 1 diabetes results from the selective autoimmune mediated destruction of the insulin-producing beta cells within the pancreatic islets of Langerhans. This loss inevitably leads to changes in islet architecture and is important since in vitro studies [1] suggest that spatially dependent cellular interactions are critical for the functional coordination of islet hormone secretion. Thus, any changes to spatial configurations within islets may contribute to the defects in hormone secretion observed in individuals with type 1 diabetes. Moreover, disruption of islet architecture may also lead to increased islet cell stress, which could in turn promote immune cell recruitment and further augment beta cell loss. Therefore, the quest to gain an improved understanding of islet architecture in humans, in both health and disease, is paramount.

**Fig. 1:**
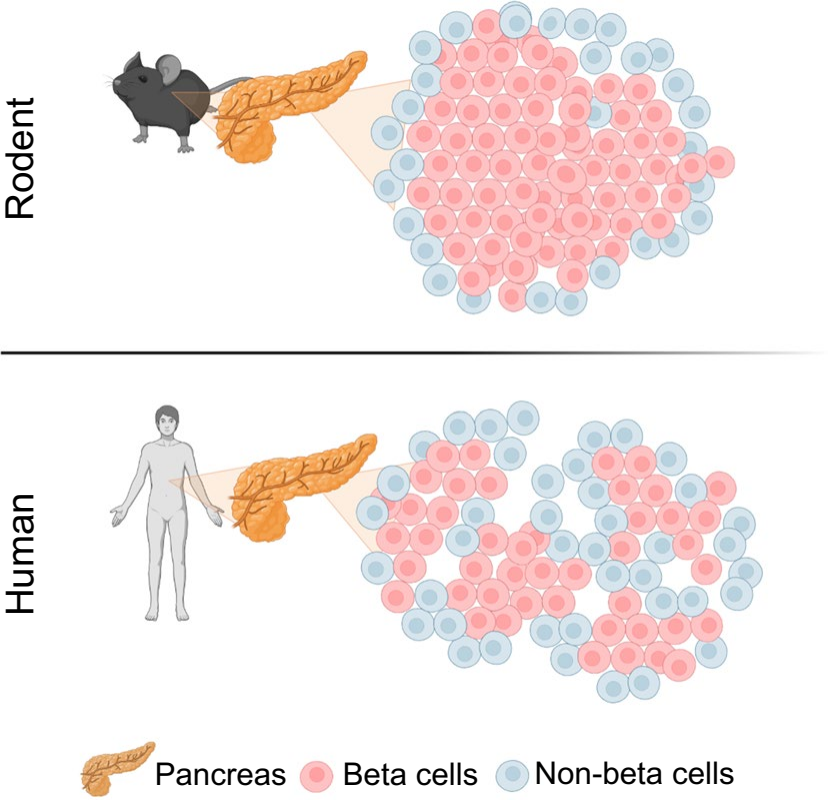
The existence of an M-C islet structure in rodents is undisputed. However, the field remains divided as to the existence of an organised M-C arrangement in adult humans. Created with BioRender.com

Elegant analyses of islet structure can be traced back to the 1930s [2] but the cellular organisation of islets became a subject of more careful examination following the discovery of somatostatin [3]. Subsequent work implied that rodent islets consistently display a striking mantle–core (M-C) architecture wherein beta cells are preferentially localised within the core of the islets, surrounded by a mantle of ‘non-beta’ endocrine cells (including alpha, delta, epsilon and gamma cells; see Fig. 1). The presence of the same architecture in human islets is less obvious from 2D-histological analysis and has long been debated.

Measurements of islet volume suggest that the range of islet size is relatively conserved across most mammalian species [4] and that the M-C structure may be common among many species, although whether this extends to human islets remains unclear [5]. Those defending the view that the M-C structure is a robust organisational feature of human islets argue that the presence of folds in the surface of the islet (Fig. 1) allows the retention of a basic M-C orientation whereas others oppose this proposition [6, 7].

One key reason that this debate is still unresolved is that relatively few human pancreatic micrographs have been examined quantitatively, in detail [8], to decide the cellular architecture. Where this has been studied, the outcomes have relied heavily on a subjective interpretation of the data [7]. With the emergence of increasingly advanced staining and spatial biology techniques, combined with digital pathology tools incorporating machine learning (ML) [9], it is now possible to analyse much larger numbers of islets in a systematic manner to interrogate the spatial relationships between the different cell types more forensically.

In the present study, multiplex staining of five endocrine hormones was visualised in islets and studied in situ by whole-slide multi-channel image acquisition and analysed with advanced image-analysis technologies. Supervised ML methods were used to efficiently extract relevant data from the whole-slide multiplex images, after which mathematical modelling techniques were used to quantitatively assess the evidence supporting the presence of an M-C structure, enabling a detailed histological study of the architecture of human islets.

## Methods

### Study population

Single 4 µm whole-slide sections of formalin-fixed, paraffin-embedded (FFPE) human pancreatic tail were studied from ten individuals: four organ donors without diabetes from Breakthrough T1D’s network of Pancreatic Organ Donors with Diabetes (nPOD) biobank (https://npod.org/about/who-we-are/); and six living donors who underwent pancreatic tail resection within the Diabetes Virus Detection (DiViD) study [10] (see electronic supplementary material ESM Table 7). Sex was self-reported and the ratio of male to female participants was 1:1 for type 1 diabetes and control cases. The two groups were also age-matched, with the mean ages (type 1 diabetes, 29.68 years; control, 28.83 years) differing by approximately ±0.85 years.

### Ethics statement

Samples from the nPOD collections are collected, held and shared in accordance with federal guidelines for organ donation and the University of Florida Institutional Review Board. Ethical approval for the DiViD study was awarded by the Norwegian government’s Regional Ethics Committee and written informed consent was obtained from all participants. Each donor (and/or their family) gave informed consent for their organs to be used in research. Those responsible for the collection of the pancreases, and creation of the biobanks, were approved by the relevant and responsible ethics committees (or Institutional Review Board), which in turn also approved the current investigations and the material transfer to the investigators.

### Tissue processing and imaging

Sections were stained using standard histological techniques employing OPAL TSA-amplification and fluorescent reagents according to the manufacturers’ guidelines (Akoya Biosciences, CA, USA; catalogue no. NEL811001KT).

In brief, samples were dewaxed using Histoclear (SLS, UK; catalogue no. NAT1330), underwent serial rehydration (100%, 90%, 70%, 50% EtOH and H_2_O alone) and 20 min of heat-induced epitope retrieval in citrate buffer (pH6) (Sigma-Aldrich). Sequential rounds of immunostaining followed, with validated antisera specific for all five islet-endocrine hormones (for full details of suppliers see ESM Table 8). A ductal cell marker (CK19) was also included (see ESM Table 8 for details). An antibody-negative slide was generated to provide an example of endogenous pancreatic autofluorescence, ensuring signal specificity during image processing.

### Image analysis

#### Image generation

Sections were imaged at 40× magnification, in seven channels, using the PhenoImagerHT slide scanner (Akoya Biosciences), whereby contiguous images (‘tiles’) were captured across the entire section and tagged with (*x*,*y*)-coordinates. Each tile was spectrally unmixed using the proprietary InForm Software v2.6 (Akoya Biosciences) to distinguish true signal from autofluorescence and ‘bleed-through’ from neighbouring channels.

Processed image tiles were stitched together using QuPath, an open-source bioimaging analysis software [11]. The resultant pyramidal images facilitate exploration of the tissue at a range of magnifications. However, the large file size of the stitched whole section, high-resolution images made conducting analysis on a standard personal computer impossible. This issue was overcome by creating smaller sub-images of the whole section. Critically, the (*x*,*y*)-coordinates of each tile retain the relative spatial coordinates of the whole section, allowing whole-slide information to be calculated.

**Fig. 2:**
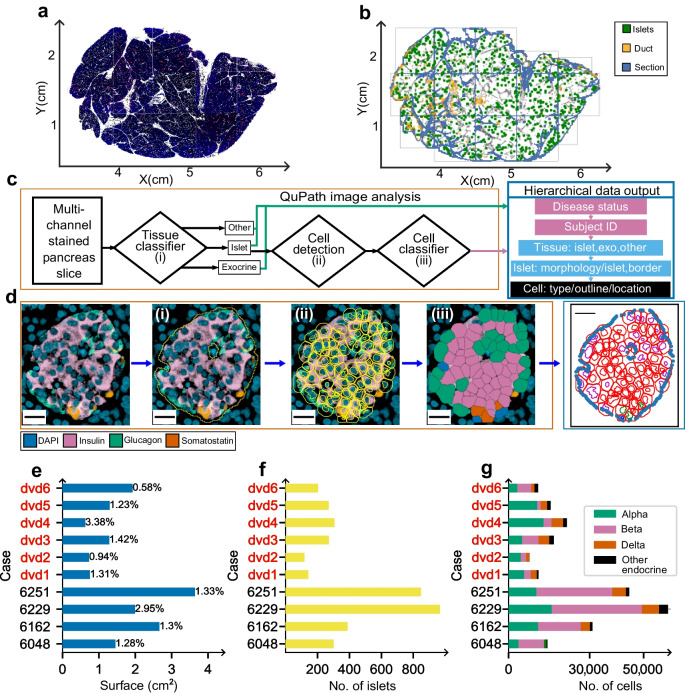
Image-analysis pipeline. (**a**) Representative example of a whole section image of a stained human pancreatic section created by the stitching of contiguous tiled micrographs (control case 6251). (**b**) Illustrative example of detections created during image-analysis pipeline of the entire section including locations of exocrine borders, islets and ductal regions. (**c**) Steps of the image-analysis pipeline in QuPath. Elements and processes (e.g. classification) are represented with rectangles and rhombi, respectively. Through three different classification processes (i–iii, described in **d**), images are turned into data (blue box) and exported into a hierarchical data structure. (**d**) Example of the pipeline presented in (**c**), applied to a region of the pancreatic tissue. Each inset illustrates the visual output of the classification processes. The first inset shows an image of a representative islet. The rest of the insets overlay the detections (i, classification of islet; ii, detection of cells within the islet; iii, classification of cells). The final inset shows the reconstruction of the islet using only the extracted data. Scale bar, 50 µm. (**e**–**g**) Scope of data. The vertical bar plots show, for the ten individuals considered (control in black and type 1 diabetes in red), the total surface area of the tissue analysed (including the percentage of tissue corresponding to islets) (**e**), the total number of islets (**f**) and the total cell count by endocrine cell type (**g**)

Figure 2a shows a representation of the whole slide for control case nPOD6251 as an exemplar of where sub-sectioning was required (Fig. 2b), where the data extraction pipeline (Fig. 2c, d) provided a summary of endocrine cells identified (Fig. 2e–g), each associated with a centroid-(*x*,*y*)-coordinate. Further technical details are provided in ESM Methods (Image acquisition and analysis). See also the expanded version of the pipeline in ESM Fig. 1.

#### Tissue classification

Using QuPath, a pixel classifier was trained to segment each pixel into tissue subtypes by supervised learning (with set parameters described in ESM Methods, ESM Table 1). Using representative samples of the images from all studied individuals, the investigator provided the software with examples of glass (no tissue), exocrine tissue, duct and islet regions (see Table 1).

**Table 1:** Tissue subcategories considered in the pixel classification

Tissue type	Characterised by pixel with the property:
Glass/background	No staining
Exocrine tissue	Positive for DAPI but negative for all hormones
Duct (CK19^+^ marker)	Positive for CDK19^+^ marker
Islet	Endocrine-hormone positive

Training regions were created by hand and Fig. 2b illustrates the tissue classification process over the whole section. Endocrine cell clusters >1000 µm^2^ (~five cells) were defined as islets. Structures smaller than this threshold typically correspond to endocrine clusters too small to meet an M-C structure and were therefore outside the scope of this investigation. Further details are provided in ESM Methods: Tissue classification.

#### Cell detection and phenotyping

Employing the DAPI channel within the regions classified as islets, QuPath’s ‘cell detection algorithm’ was used to define regions corresponding to the cell nuclei and to estimate the location of the plasma membrane. Thresholds based on user-defined parameters such as cell size, and nuclear and hormone staining coverage, were used to approximate nuclear and cellular boundaries (see ESM Methods: Cell detection and ESM Table 2 for details). An endocrine cell subtype classifier was then created by iterative training of the software to differentiate between each of the five endocrine lineages of segmented cells in a subset of islets (see ESM Methods: Cell classification and ESM Tables 3, 4 for details). Approximately 6% of cells within islets were not labelled by any of the five hormones (designated as ‘none’) and were excluded from the analysis. The accuracy of the detection and classification algorithms was iteratively tested by consulting with an experienced pancreatic histologist whereby the algorithm output was checked on five separate occasions employing a small but representative sample of the images under study. Strong agreement was reached.

Application of the trained detection and classification algorithms then allowed rapid (in the order of minutes) processing of the large image set.

#### Data extraction and analysis

The classification and detection process yielded a set of ‘closed curves’ corresponding to the boundary of each object of interest (e.g. the contour/outline of a cell nucleus or islet). Each curve possessed metadata describing the object type (e.g. islet or cell). Figure 2d shows a graphical ‘reconstruction’ of the islet using only the detected curves, with the blue dots corresponding to the boundary between islet and exocrine tissue. The smaller closed curves correspond to cell nuclei within the islet, with colours representing the cell type assigned by the cell classifier. The information in these curves is organised in a hierarchical manner, as illustrated in Fig. 2c, giving linked identity and localisation information for every pixel, cell and islet. Exporting data from images is further discussed in ESM Methods.

QuPath allows these curves to be exported directly with the possibility of exporting tables containing all the associated relevant information from the curves. The extracted data described in Table 2 were then considered.

**Table 2:** Description of the data exported from QuPath

Data name	Data type	Description
Islet boundary	Closed curved	Points marking the boundary of each islet
Nuclear boundary	Closed curved	Points marking the boundary of each cell nucleus (only endocrine cells)
Islet data frame	Table	Table in which each row contains information about an individual islet Properties (columns) include case, area, centroid, no. of cells detected corresponding to each cell type
Cell data frame	Table	Table in which each row contains information about an individual cell Properties (columns) include area, centroid of the nucleus, islet id they belong to, mean and SD of the concentration of the different channels

Each row corresponds to a file

### M-C structure investigation

**Fig. 3:**
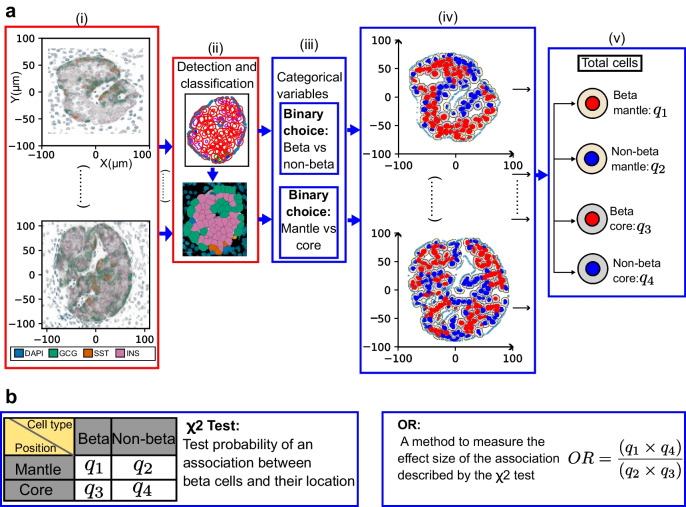
Statistical testing of the M-C structure considering large groups of cells. (**a**) Previously classified endocrine cells were assigned into two categories: beta cells (red nuclei) and non-beta cells (blue nuclei). Based on distance to the islet ‘border’, all these cells were placed into either the mantle (yellow ‘cytoplasm’) or the core (grey cytoplasm) category. The vertical ellipses in (i) and (iv) illustrate that this process was performed for the whole population of islets in the study. (**b**) Counting the total number of cells belonging to each category, a contingency table was created to perform a χ^2^ test for independence, and strength of association was tested via the OR. This protocol was repeated in all sub-groups described in ESM Table 6. GCG, glucagon; INS, insulin; SST, somatostatin

To investigate the existence of an M-C structure in human islets, a mathematical criterion was established to determine whether beta and non-beta endocrine cell types occupy a mantle or core position (see ESM Methods: Classifying cells in the islets as mantle or core for details). Thus, each positively stained cell within an islet was classified as a beta cell (Fig. 3a(iv): red nuclei) or non-beta cell (blue nuclei).

In addition to being classified by cell type, the minimal distance (d_min_) between the contour of each cell nucleus (i.e. nuclear boundary in Table 2) and the border of the islet to which the cell belongs (blue line in Fig. 2d) was also computed. If this distance was smaller than d_min_=8 μm, the cell was labelled as mantle (illustrated by a yellow ‘cytoplasm’ in Fig. 3), otherwise as core (grey ‘cytoplasm’). The distance threshold was chosen to ensure that only the first neighbours to the islet border are considered as forming a mantle. The d_min_ was tested at a range of values to ensure that the chosen distance of 8 μm was robust against small changes in the classification of the border. Consequently, the population of cells was endowed with two binary variables corresponding to the attributed cell type and its position in the islet (see Fig. 3a and ESM Methods: χ^2^ test for independence between the cell type and position, ESM Table 5]).

#### Analysis 1: association between cell position and type

The χ^2^ test for independence was chosen to explore the potential association between cell type and position, and the effect size was measured using the OR (Fig. 3b). (For further details, see ESM Methods: χ^2^ test for independence between the cell type and position. In particular, ESM Fig. 2 shows how the *p* value is affected by the sample size while the effect size remains constant.)

### χ^2^ test of independence for groups of cells belonging to different islet size and disease status

To investigate the relative impact of potential confounders that may influence the dependence between cell type and position in the total population, the χ^2^ test for independence was carried out in nine sub-groupings of cells (outlined in ESM Table 6).

#### Analysis 2: quantifying the M-C structure at islet level

Employing the binary variables from the previous section (Fig. 3), the organisation of islet cells was studied. The configuration of each islet was thus modelled using the cell counts described in Table 3.

**Table 3:** Islet cell counts used to fully characterise a given islet

Symbol	Quantity description (per islet)
N	Total number of cells
NB	Number of non-beta cells
M	Number of cells in the mantle
NBm	Number of non-beta cells in the mantle
C	Number of cells in the core
B	Number of beta cells

From these numbers, the probability, *r*, that at least the observed number of non-beta cells in the mantle of a given islet (NB_m_) occurred due to random arrangement was computed. The closer to *r*=1, the more random the arrangement of cells, whereas the smaller the value of *r*, the stronger the evidence supporting the M-C structure for the islet under study. The *r* value was computed for each islet in the dataset. For further details, see ESM Methods: Quantification of the M-C structure at islet level, including ESM Fig. 3. Interactive tools for calculating the *r* values can be found in ESM Table 11 in the interactive spreadsheet.

#### Digital siblings: a population of synthetic islets with known random spatial arrangement

**Fig. 4:**
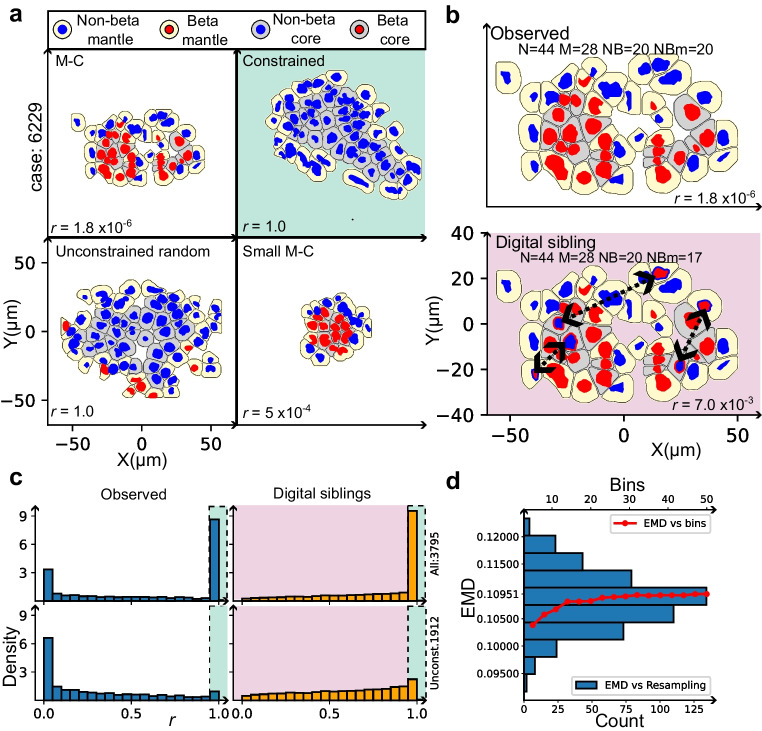
Quantifying the M-C structure at islet level. (**a**) Four representative examples of real islets reconstructed from QuPath detections. The *r* value quantifies the level of randomness of each islet architecture. Colour conventions are as for Fig. 3. An example of a constrained islet, that cannot meet the M-C structure, is highlighted in light green. (**b**) An observed islet was randomly and digitally rearranged to generate a version of itself, called a ‘digital sibling’, by swapping beta cells in the core for non-beta cells in the mantle (highlighted in light red). (**c**) Histograms of the *r* values were generated for the observed (blue histogram) and digital sibling (orange histogram bars/light red background) populations, considering the whole population of islets and after the removal of the constrained islets. (**d**) Standard graphical representation of the EMD between the distributions of *r* values obtained from unconstrained islets belonging to the observed and digital siblings populations (blue and orange histograms, respectively, in **c**). Blue bars (in **d**) show the distribution of values of the EMD (*y*-axis) when *r* values are resampled (with repetition, counts on bottom *x*-axis). The red line shows the EMD as the number of equidistant bins for *r* is changed (top *x*-axis)

To assess if the distribution of the *r* values in our cohort differs from a situation where the cell types are known to be randomly organised within the islets, a second population of ‘digital sibling’ islets was created. Each digital sibling was generated by taking a given islet, retaining its morphology (e.g. islet boundary location) and the proportions of its classified cell types (described in Table 3), and then computationally reallocating these cell identities into randomly generated M-C configurations. A simple example is illustrated in Fig. 4b, where a ‘digital sibling’ was created from a real, observed islet in which three cells were randomly swapped with three other randomly selected cells. In this example, these were core-beta cells and happened to be ‘swapped’ (see dotted arrows) with non-beta mantle cells to create a randomly shuffled version of the true configuration. The distribution of the *r* values for the population of digital siblings was computed.

Thus, the ‘sibling population’ provides a baseline dataset of *r* values associated with known but random architectures with which to compare the observed islet configuration in our participants.

#### Constrained islets

It is important to note that it is impossible to meaningfully classify some islets as having an M-C structure, either due to their innate morphology or because of the cell types (beta vs non-beta) present. We refer to the general subpopulation of islets that cannot be meaningfully classified as having M-C structure as ‘constrained islets.’

Figure 4a illustrates an example (shaded green) of a constrained islet where, because only non-beta cells are contained therein, only one arrangement is possible. These islets necessarily have an *r* value of 1. More specifically, constrained islets were defined as those islets possessing fewer than three:

- beta cells
- non-beta cells
- core positions

Unconstrained islets were the focus of the analysis in this stage of the investigation. Further details are given in ESM Methods: Unconstrained and digital siblings islets.

#### Estimating the effect of the M-C structure using the earth mover’s distance

The distribution of *r* values for the observed (unconstrained) islets was compared against the distribution obtained in the digital sibling population, using a metric known as the earth mover’s distance (EMD) [12]. In essence, the EMD provides a numerical measure of the effort needed to transform one (the target) data distribution into another by moving and reshaping it to recreate the target distribution in the most efficient manner, comparable with how one might reshape one ‘landscape’ to another by moving the smallest amount of ‘earth’ over the shortest distance. By understanding the optimal redistribution needed for this transformation, the EMD highlights similarities and distinctions between datasets.

EMD is robust against resampling of the data and/or changes in the number of islet bins, as illustrated in Fig. 4d, in which computations of the EMD were carried out for a different number of bins (binned by the value of *r*) and resampling the data (random rearrangements of sibling islets). The bins were considered equidistant and the EMD was normalised such that its values are between 0 (identical distributions) and 1 (maximum discrepancies) per metric included in the comparison. The latter occurs when the work required corresponds to that which is necessary to ‘move’ all of the ‘earth’ from one extreme of the distribution to the opposite. A mini-tutorial is included in the ESM (Earth mover’s distance mini-tutorial) to provide more insight into this method.

#### Analysis 3: quantifying the relationship between islet size and beta cell fraction for islets

**Fig. 5:**
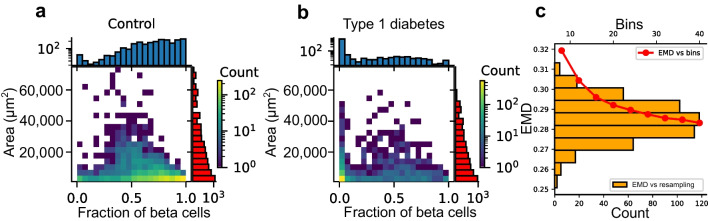
Distribution of islets as a function of two key variables / islet. (**a**, **b**) Bidimensional histograms, plotting the distribution of islets as a function of the fraction of beta cells (x-axis) and the area of each islet (y-axis) are shown for the control (**a**) and type 1 diabetes (**b**) cases. (**c**) The EMD between (**a**) and (**b**) was computed by resampling the data (orange bars in histogram) and by changing the number of bins. A well-defined distance is observed

The utility of the EMD extends to an ability to distinguish several features within distributions gleaned from multivariable datasets. Therefore, for completeness, the distribution of islets as a function of the fraction of beta cells vs the area of islets was computed for islets belonging to control and type 1 diabetes cohorts and is shown in Fig. 5a and b, respectively. The distance between the distributions corresponding to the control and type 1 diabetes islets was also measured using the EMD and its robustness was studied by changing the number of bins (ranging from 8 to 40) or resampling the data (500 times) (Fig. 5c). The bins were considered equidistant in both *x*- and *y*-axes (in Fig. 5a, b), and the EMD was normalised such that the maximum distance along each axis was 1. Consequently, the maximum possible work corresponds to 2 in this analysis.

## Results

### Islet and cells computation (Analysis 1)

In this study, 3518 islets (comprising 252,756 cells) were detected: 107,135 cells were classified as beta cells; and 145,621 were classified as non-beta cells. Of the cells, 148,809 were nominated as mantle vs 103,947 as occupying a core location. Restricting the attention to the control cases at islet level, the fraction of beta cells peaks at 80%, in good agreement with earlier observations (see Fig. 5a). The fraction of alpha cells peaks at <5%, particularly in smaller islets (<10,000 μm^2^), which represent around 30% of the total islet population. This observation corresponds with recent reports [13].

### χ^2^ test of independence between cell type and position

**Fig. 6:**
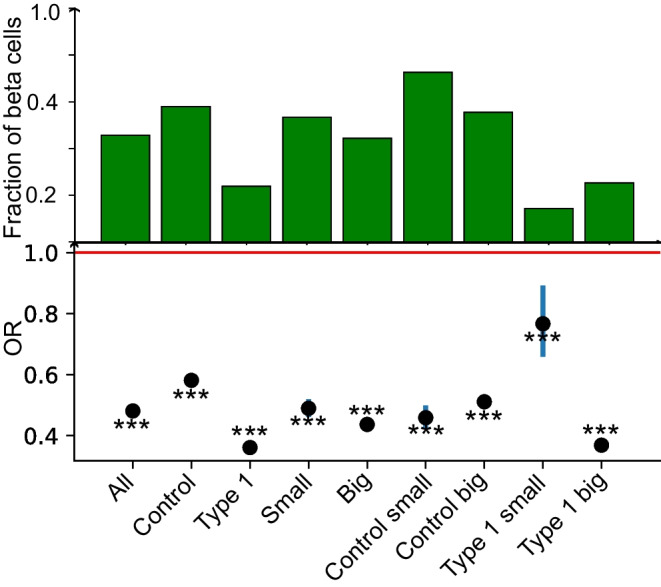
OR comparing the fraction of beta cells with islet size, and the impact of type 1 diabetes (‘Type 1’). The fraction of beta cells in each group is shown together with the median of the OR (black dot) and the 95% CI (blue lines). ***p<0.001

When considering cell identity and position within islets and using the categorical variables defined above, the χ^2^ test for independence yielded statistically significant *p* values at a threshold of α=0.001, supporting the existence of a relationship between cell type and position. This was the case for each of the nine groups considered, specified in ESM Table 6. However, the *p* value does not provide information on the type of dependence. To understand the direction and magnitude of the relationship between cell type and location, the OR was considered. Importantly, OR is not impacted by sample size, and while cells from the smaller type 1 diabetes islets showed more variability, all islet groups adhered to the M-C structure. For each group, the OR was <0.8<1 (an OR below one supports adherence to the M-C hypothesis). The OR results are summarised in Fig. 6. CIs were computed by resampling (with replacing) the data 500 times (see ESM Methods: χ^2^ test for independence between the cell type and position for further details).

Therefore, when the population of islets is considered overall, endocrine cells defined as beta or non-beta show a propensity to be located either in the islet core or mantle, respectively, supporting the hypothesis that a native M-C structure occurs in adult humans when islets are >1000 µm^2^ in cross-sectional area and regardless of type 1 diabetes disease status.

### M-C structure quantification at islet level (Analysis 2)

Among the 3518 islets analysed, 1912 were considered as unconstrained when examined in two dimensions.

**Fig. 7:**
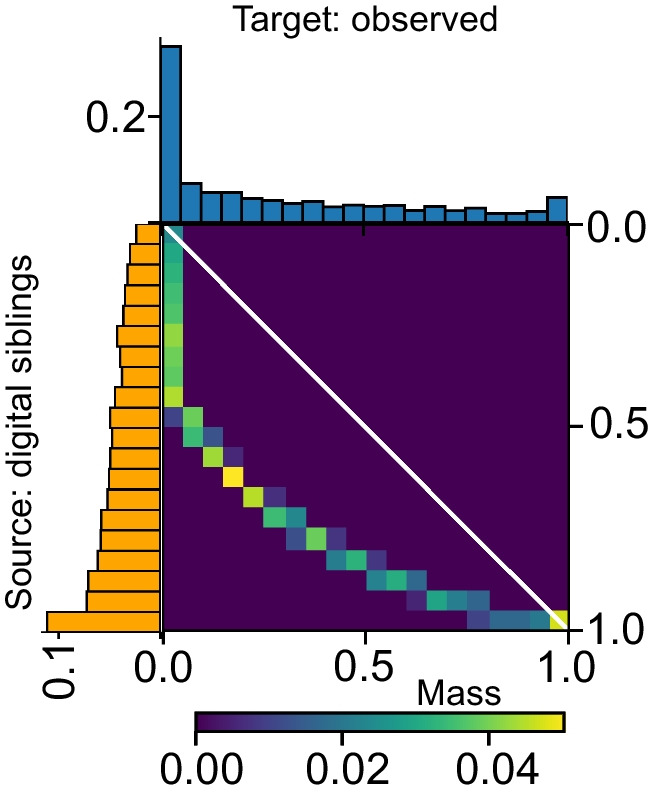
Graphical illustration of transport plan for the EMD in Analysis 2. The histograms (orange, digital siblings; blue, observed islets) illustrate the frequency distributions of *r* values (in bins) associated with the calculated probabilities that given islets in the observed and digital sibling populations adhere to an M-C structure, where *r*=1 represents no likelihood of M-C adherence. The EMD provides a ‘transport plan’, illustrated by the heatmap matrix, describing how much ‘work’ is required to ‘transform’ the ‘digital sibling’ populations to be more like their ‘observed counterparts’. The white diagonal line represents the point where the distributions would be identical, and no work would be required to transform one bin to the other. Purple indicates low level transformation is needed, while yellow indicates a large transformational cost. Thus, the biggest differences in the distribution curves considered here are revealed by the degree of skewing towards the smallest values of *r* in observed islets

As expected, in the ‘digital sibling’ distribution (Fig. 7; orange histogram), many of the islets yield an *r* value at, or close to, 1 and the distribution is relatively flat as might occur through iterative generation of randomly organised ‘sibling islets’. However, in the observed islets, the sample shows a pronounced peak (Fig. 7; blue histogram) at the lowest values of *r*, highly suggestive of strong adherence to the M-C structure. This is in keeping with the findings in Analysis 1. This qualitative observation can be quantified by means of the transportation plan (see Fig. 7), which illustrates how the *r* values of the source distribution (digital siblings, yellow histogram) should be redistributed to obtain the distribution of the observed islets. As can be appreciated, all of the non-zero entries in the transportation plan lie below the diagonal (white line in Fig. 7), suggesting that in comparison with the base case (digital siblings), the observed *r* values are strongly skewed towards smaller values of *r*. This observation is in good agreement with the M-C hypothesis.

To quantify differences between the distribution of *r* values pertaining to M-C structure in observed (real) vs digital sibling (randomly organised) islets, the EMD was calculated as emd-1d=0.1 (emd-1d is the comparison of distributions examining one metric [in this case *r* values], and if emd-1d=0, there is no difference in the distributions). These results confirm that in adult human islets that have sufficient components to form an M-C structure, a random, unstructured organisational pattern of endocrine cells is unlikely.

### Contrasting the distribution of islets in control and type 1 diabetes cohorts (Analysis 3)

The EMD was also employed to measure the distance between the bidimensional distributions of islets when comparing the type 1 diabetes and control cohorts. This is the distribution of islets as a function of both their fraction of beta cells and areas. The distance was found to be emd-2d=0.285 (0<emd-2d<2), where a distance of 0 means no discernible differences in the distribution landscapes of the included parameters.

The EMD confirms that there are clear differences in beta cell fraction between type 1 diabetes and control islets (see Fig. 5). Furthermore, when considering the optimal transport plan, the most marked differences between the bidimensional distributions were found to be along the axis of fraction of beta cells in the smallest islets.

## Discussion

The notion that islet architecture adopts an M-C configuration in rodents is well-established but there remains no definitive consensus regarding the architecture of human islets. Despite the growing consensus that an islet architecture is closely linked to function, there have been relatively few attempts (e.g. [5–8]) to categorically characterise the cellular arrangement of human islets, and most do not adopt a mathematical approach to quantification.

Therefore, the primary goal of this work was the development and application of a novel and shareable analysis pipeline that can be employed to quantitatively assess the features of islet architecture in histological sections of pancreas in a reproducible way. By employing this pipeline, we confirm the presence of an M-C structure in human adult islets, in both non-diabetic individuals and adults with recent-onset type 1 diabetes. We also describe a more rapid decline in the beta cell fraction in smaller vs larger islets in adult recent-onset type 1 diabetes.

### A novel pipeline to assess islet architecture quantitatively

When histological sections are viewed in two dimensions, the confounding presence of ‘empty’ vascular spaces (frequently described in human islets but more rarely in rodents) may confound the use of Gestalt principles [7] to classify the complex islet structures found in humans, especially when compared with the apparent homogeneity of rodent islet architecture [5]. It is also likely that the dearth of adequate tools and sufficiently large datasets has impeded progress in addressing this conundrum in human pancreas [8].

Modern imaging and open-source software facilitate the generation of large datasets, and ML techniques then offer the capacity to study large numbers of histological samples in robust and reproducible ways.

Multidisciplinary practices designed to leverage the strengths of these advancements is becoming the gold standard in research, and we have combined these technologies to develop quantitative methods that enable us to statistically test more definitively the existence and nature of an M-C architecture in human islets. The pipeline and techniques described here are readily transferable and have wide applications within the field of spatial biology.

### Examination of M-C structure in human islets

Employing two complementary approaches, we conclude that the M-C structure is conserved in adult humans regardless of disease status. As might be expected from the cellular rearrangements that inevitably accompany beta cell loss, we also note that the M-C structure is less obviously conserved (higher OR) in smaller type 1 diabetes islets (Fig. 6). We also demonstrate that smaller islets contain a reduced fraction of beta cells (see Analysis 3; Fig. 5). This means that the estimate of OR has higher variability, as indicated by the wider CI (see Fig. 6). However, statistically (and, perhaps, surprisingly), such islets still remain highly adherent to an M-C architecture. The present results confirming a clear M-C structure in human islets add weight to the hypothesis that alpha cells may be arranged spatially to shield beta cells [8] from the extra-islet environment by virtue of their preferential localisation at the islet periphery and close to the vascular spaces.

This shielding within the M-C structure is important since it has been demonstrated that some islets isolated from the resected pancreas of the DiViD study participants could be reinvigorated towards glucose-responsive insulin secretion following a period of tissue culture [14]. It is unclear what drives this functional recovery but, as our data also reveal that the M-C structure is retained in these individuals, it is tempting to hypothesise that those islets that retain an M-C architecture and survive the immunological onslaught offer the potential that endogenous insulin secretion might also be recovered in vivo, if the M-C structure is maintained, and the diabetogenic environment and associated autoimmunity is controlled effectively.

While we demonstrate that the M-C structure holds true even as beta cells are lost in adult-onset type 1 diabetes, it is important to note that similar conclusions cannot be automatically extrapolated to the islets of children recently diagnosed with type 1 diabetes. This is because in young children beta cell mass is lost much more extensively and rapidly than in teenagers and adults [15]. Further work will therefore be required to assess the architectural organisation of residual insulin-containing islets in individuals diagnosed at earlier ages or who were autoantibody positive but without diabetes, compared with age-matched control individuals.

### Implications for islet transplantation

It is interesting to note that some evidence implies that less-purified human islets (i.e. those that are less well ‘digested’ during the isolation process) may retain better insulin secretory function [16] than islets subjected to more rigorous digestion during isolation. The reasons for this are unknown but could reflect the maintenance of a more ‘intact’ islet architecture. While the ‘folded’ architecture of human islets may partially protect against total non-beta cell ablation during islet isolation, the selective loss of peripheral non-beta cells that still occurs may contribute to the early loss of function of transplanted islets [17]. Application of the present methods to assess islet architecture after the isolation process could, in future, help to evaluate this proposition.

Extending this reasoning, with an increasing push to develop functionally effective ‘pseudo-islets’, made either by reaggregation of dispersed human endocrine cells, or via induced pluripotent stem cell (iPSC)-derived methods, we suggest that the provision of a quantitative tool with which to assess the architectural features of these maturing islets may also provide an added means to evaluate and improve the functionality of such pseudo-islets.

### Pancreatic development over the lifetime

Additionally, the pipelines utilised here are ideally suited to studies designed to further the understanding of how the architecture and cellular composition of islets change through normal development in the human pancreas. It has been proposed that early environmental cues may impact the islet cell populations and their distribution [18]. We hereby offer an accessible tool to study the architectural trajectory of maturing islets in existing pancreatic biobanks.

### Utility of the pipeline in other research domains

Our pipeline also offers utility for studies focusing on the structural architecture of islets in monogenic forms of diabetes and in type 2 diabetes. Moreover, it may also offer insights into the still poorly understood changes in islet architecture that lead to cystic fibrosis-associated diabetes since this often occurs later in the progression of the condition, implying a gradual loss of islet integrity. The impact of ageing and other diseases of the pancreas, including endocrine tumour development, are also ripe for study.

### Disease heterogeneity

This present work was carried out considering single sections from the tail region of pancreases from ten white European adults. It is increasingly accepted that there is considerable heterogeneity in the human pancreas, perhaps caused by environmental cues experienced during development. It will thus be important to extend the analysis to additional racial and ethnic groups, at different ages and to a larger sample size. Further, despite the 1:1 sex ratio among our study participants, to explore if any differences in islet architecture in health and/or in response to type 1 diabetes onset occur between sexes it will be important to increase our sample size.

### Expanding the quantitative toolkit in spatial biology studies

The inherent bidimensional nature of the present study opens the question as to whether the results of this work could be generalised to three-dimensions. A recent groundbreaking study [13] has expanded our understanding of the islet and its heterogeneity; the authors meticulously sectioned, stained, imaged and rebuilt one entire pancreas, showing that it is possible to image the whole organ in three spatial dimensions. A natural next step would be to test our M-C hypothesis with these data.

However, for the majority of situations where study is limited to 2D sections, this model potentially also offers a tool (not dissimilar to elegant work carried out using electron micrographs to model the location of lipofuscin bodies in beta cells [19]) for ensuring that when islets are described as, for example, insulin deficient, it is possible to estimate the likelihood that this statement holds true throughout the 3D structure. It will be interesting to examine the M-C structure in other regions of the pancreas.

Thus we offer a quantitative and robust opportunity to adapt these protocols to explore many other aspects of islet composition and structure (e.g. comparing protein expression profiles in islets between individuals at different ages [with and without disease] or even comparing islets within individuals whose endocrine cells may be expressing key features of interest [e.g. inflamed vs non-inflamed islets], or islets expressing particular staining patterns [e.g. hyperexpression of immune markers] or high levels of stress).

### Refining the pipeline

The analysis presented relies strongly on the detection algorithms employed. The comparison between the output produced by the algorithm and the classification of an expert shows that the results are reliable. In a future study, a more in-depth evaluation of segmentation methods will allow a better estimate of the uncertainty in the detections and classifications. Furthermore, as we continue to expand our exploration of the aetiology of type 1 diabetes in the pancreas to include individuals at different ages and from various regions of the pancreas, stained with all five hormones (necessary for true ‘islet area’ to be assessed), and as we continue to refine and expand the pipeline, we will continue to update the field in future publications.

## Supplementary Information

Below is the link to the electronic supplementary material.

ESM1 (PDF 778 KB)

ESM2 (XLSX 14.8 KB)

## Data Availability

Data and code are available on request and can be obtained from the corresponding author.

## Abbreviations

DiViD: Diabetes Virus Detection
d_min_: Minimal distance
EMD: Earth mover’s distance
M-C: Mantle–core
ML: Machine learning
nPOD: Network of Pancreatic Organ Donors with Diabetes
