# Supplementary material for: Beyond the loss of beta cells: a quantitative analysis of islet architecture in adults with and without type 1 diabetes
Source: Diabetologia. 2025 Feb 26;68(5):1031–43. doi: 10.1007/s00125-025-06376-9 (PMC12021988; doi:10.1007/s00125-025-06376-9)
Supplement: Supplementary file 1 — ESM1 (PDF 778 KB) [file 125_2025_6376_MOESM1_ESM.pdf]

Electronic supplementary material of the manuscript: “Beyond the loss of beta cells: A quantitative analysis of islet architecture in adults with and without type 1 diabetes”

Methods

Image acquisition and analysis

The collection of tiles generated, following the procedure described in the subsection *Tissue processing and Imaging* of the manuscript, is the starting point of the image analysis carried out using the software QuPath.

Sub-images of the whole slide were generated using Peter Bankhead’s stitching script [https://gist.github.com/petebankhead/b5a86caa333de1fdcff6bdee72a20abe] in QuPath. For each case, the number of sub-images is dictated by the available memory of the computer undertaking the analysis. All the sub images in the study were included into a single QuPath project, where the training, detection and classification of tissue and endocrine cells were performed. The image analysis in QuPath can be pictured as a series of procedures (represented by rhombi) that convert objects (rectangles) into new objects, as illustrated in ESM Fig. 1 (an expanded version of the pipeline presented in the main text). The red and blue rectangles enclose the parts of the image analysis occurring in the QuPath project, and the data exported, respectively. The roman numerals below each procedure coincide with those in the inset of the Fig. showing the pipeline in the manuscript. *The classifiers are available upon request.*

In what follows, a more detailed description of the classification processes is presented.

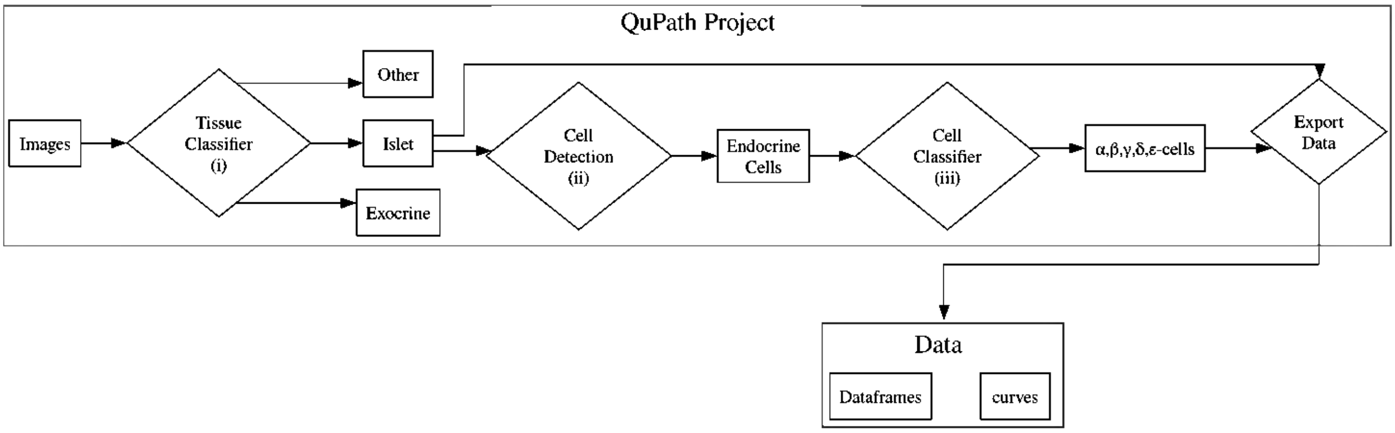

ESM Fig. 1: *Expanded version of the pipeline presented in Fig. 2* ***c****. Every rectangle corresponds to an object and every rhombus to a procedure.*

Tissue classification

Considering a small but representative sample of the images for training purposes, regions of these sample images were labelled by an expert in our team into the categories presented in Table 1 of the main text. These annotations were employed to train the *pixel classifier* within QuPath. *The Artificial Neural Network Multilayer Perceptron (ANN MLP)* was the classifier considered, with the specifications shown in ESM Table 1.

ESM Table 1: *Parameters used for the Tissue classification in QuPath.*

Field	Value
Input	All eight channels
Scale	Original (1x)
Resolution	2 [μm/pixel]
Filters	Gaussian and Laplacian
Minimum Area for islet	1000[μm^2]

Applying the tissue classifier to the images in the study, a segmentation of the images into the above-mentioned classes was obtained. In practice, the classified tissues in QuPath are the collection of closed curves corresponding to the boundary between any two classes of tissue. In Fig. 2 of the main text, panel **d** (i) illustrates the boundary between the islet and the exocrine tissue. Similarly, panel **b** shows these boundaries over the whole slide. High-level quantities of these curves, such as the area they enclosed, their perimeter, curvature, and the maximum, minimum, and standard deviation of each channel are also provided by QuPath (see Table 2 in the manuscript).

Cell detection

The built-in cell detection routine in QuPath was performed within the pixels classified as islets. The routine, based on geometrical properties (e.g. maximum/minimum areas, threshold, and width) of the stains of DAPI channel, does not require any training. Instead, its output is fully determined by the parameters presented in ESM Table 2.

ESM Table 2: *Parameters for cell detection within the pixels classified as Islet.*

Field	Value
Detection channel	DAPI
Requested Pixel Size	0.5 [μm]
Background radius	8 [μm]
Median filter radius	1.2 [μm]
Threshold intensity	0[μm]
Sigma	1.2 [μm]
Threshold intensity	2.0
Maximum/Minimum Area	80/4[μm^2]
Cell Expansion	5 [μm]

The algorithm also provides an estimate for the membrane of each cell detected. Starting from the contour of the nuclei, the estimation of the membrane expands in the normal direction until it either reaches a user defined distance (controlled by the parameter *cell expansion)* or collides with the estimated membrane of a neighbouring cell. An example of the detected cells is shown in Fig. 2 (of the main text), Panel **d** (ii). Each detected cell has a parent object corresponding to an islet.

Cell classification

Similarly to the Tissue Classifier, a small but representative subset of cells detected within islets was considered to label and train a cell classifier within QuPath. Representative endocrine cells were labelled by an expert into one the categories specified in ESM Table 3, according to the hormone (marker) covering the largest area in the vicinity of its nucleus. Cells labelled as none were excluded from the islet.

As in the Tissue classification, here an ANN_MLP classifier was also employed. The parameters considered for the cell classification are presented in ESM Table 4.

ESM Table 3: *Markers and cell types considered in the cell classification.*

Cell type	Marker
Alpha	Glucagon
Beta	Insulin
Gamma	Pancreatic polypeptide
Delta	Somatostatin
None	No endocrine marker was found in the vicinity of the nucleus

ESM Table 4: Parameters used in the cell classification.

Field	Value
Input	All geometric features of the nucleus provided by QuPath (e.g. Area, Perimeter, circularity, etc)
Filters	No filters
Scale	1x

Because the cell classification dealt with objects rather than pixels, there was no spatial resolution parameter. The cell classifier was applied to all detected cells in the QuPath project. Inset (iii) in panel **d** of Fig. 2 illustrates the result of the classification of cells detected in inset (ii).

Exporting data from Images

The image processing pipeline resulted in a collection of classified objects. These objects correspond to the curves marking the boundaries of these objects plus additional attributes. They are organised hierarchically. The highest level is the image itself, followed by annotations, and in the case of islets, individual cells of various types.

QuPath allows for exporting both the set of points defining these curves and relevant information about the different objects in data tables. As an example of the pipeline's capabilities, the rightmost inset of Fig. 2 Panel **d**, showcases the reconstruction of an islet using only data obtained through the described processes. The pipeline can perform the same analysis robustly on a larger scale, even when the tissue resolution is too high, and the resulting file size is too big for QuPath to load the entire image at once. Panels **a** and **b** of Fig. 2 shows this capability, with **a** showing the whole tissue section and **b** its reconstruction.

The standard Python libraries *pandas*, *matplotlib*, *numpy*, and *scipy* were used for further investigation and analysis of the exported data. Panel **e**, **f**, and **g** of Fig. 2 (main text) summarizes and quantifies basic statistics extracted from the data. All three bar graphs share a vertical axis listing the ten cases, divided into 4 control cases (shown in blue) and 6 type 1 cases (shown in red). From left to right, the plots depict the total tissue area considered in each case (with the percentage of tissue corresponding to islets displayed as a number on top of each bar), the number of islets identified, and the total number of endocrine cells found in each case.

Classifying cells in the islet as mantle or core

To investigate the tendency of non beta cells in the islet to occupy positions in the mantle (and beta in the core), a criterion to classify cells as positioned in the mantle or core was established.

As a result of the tissue classification, a set of points, corresponding to the boundary between the islets and the exocrine tissue was found. Two examples of islets boundary are presented in panel **a** (i) and (iv) of Fig. 3 of the main text. Left and right insets illustrate the superposition of all the channels and the reconstruction using the detections and classifications of QuPath, respectively. The collection of light blue points in the panels depicting the reconstruction, correspond to the boundary of the islet. For each cell in each islet, the smallest distance between any point in the curve representing the boundary of the islet and any point representing the nuclei of the cell was measured. If that distance was smaller than 8 (μm) the cell was labelled as mantle (yellow membrane). Otherwise, it was labelled as core (grey membrane). The red and blue nuclei in the Fig. correspond to beta and non-beta cells. Alternative ways to measure these distances were considered (e.g. distance between the centroid of each cell and the boundary of the islet), showing similar classification outputs.

χ^2 test for independence between the cell type and position

The pipeline provides a large set of cells with the two nominal variables *position* (values: mantle/core), and *cell type* (values: beta/non beta). This is the ideal scenario to study the possible relationship between cell *position* and t*ype* using the chi square test for independence. For a large given group of cells, the number of cells belonging to each of the four possible combinations of the two nominal categories is counted and arranged in a contingency table. Using these tables, the p-value of the chi square test for independence is obtained, measuring the probability that the two nominal variables are independent (i.e. a small p-value is evidence of a link between position and cell type). A striking feature of the results is the extremely small p-values reported (see p-values in the first tab ESM Table 10, in the accompanying interactive spreadsheet). It is known that when there is a significant link between the nominal variables, the p-value diminishes as the sample size increases, due to the test detecting smaller differences. A small p-value suggests a significant relationship between the nominal variables, but it does not provide any information about the magnitude of the relationship, nor the "direction" (the opposite of a mantle-core structure: beta cells found in the mantle and non-beta in the core would also provide a small p-value). To measure the direction and magnitude of the possible relationship, the odds-ratio (OR) was considered (see Fig. 3 **b**). The contingency table is built by counting the number of cells belonging to the categories specified in ESM Table 5.

ESM Table 5: *Entries of the contingency matrix.*

Symbol	meaning
a	Beta cells in mantle
b	Non-beta cells in mantle
c	Beta cells in core
d	Non-beta cells in core

Considering these quantities, the OR is given by:

$$OR = \frac{(a \times d)}{(c \times b)}$$

Consequently, an OR value greater than one means that the total number of beta cells in the mantle (or non beta cells in the core) is larger than the total number of beta cells in the core (non beta in the mantle) corresponds to the opposite of a mantle-core structure. Conversely, the smaller (but positive) than one the value of OR, the stronger the evidence of a mantle-core structure spatial organisation. When OR is closer to one, the effect of a possible link between cell position and type is weak.

In contrast with the p-values, the odds ratio should be independent of the sample size.ESM Fig. 2 shows the p-value (output of the chi-square test) and the OR for different sample sizes when all the cells in the group are considered.

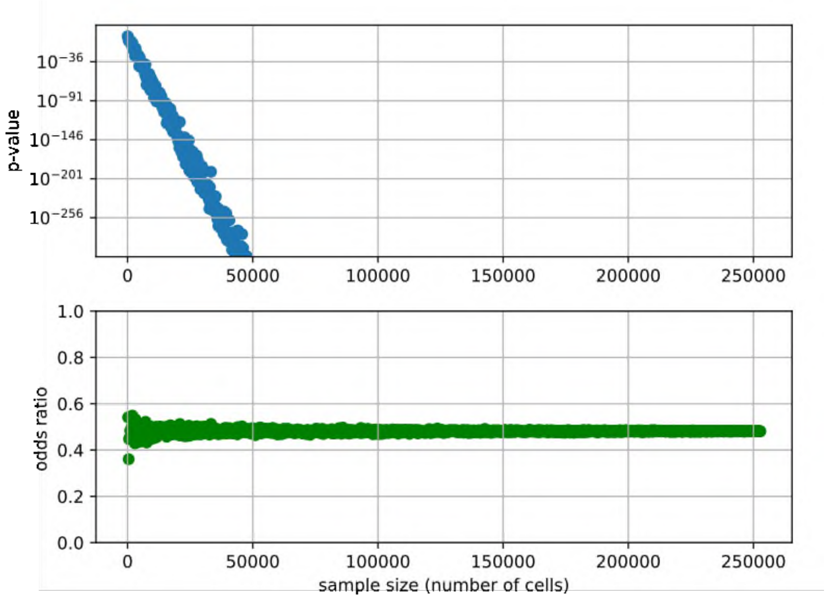

ESM Fig. 2: *Result of the chi square test for independence considering samples of the total population of detected cells. Both plots, share the horizontal axis, corresponding to the sample size in number of cells. The top and bottom plots, show the p-value (semi-log scale) and the odds ratio, respectively.*

To rule out the existence of a group of cells showing this relationship more strongly, the test was conducted in 9 groups, described in ESM Table 6.

ESM Table 6: *Cells within subgroups of islets were considered in the chi squared test for independence.*

n	Group name	Description	Islet Size threshold *(median number of cells)*
1	All	All endocrine cells in the study	None
2	Control	Endocrine cells in control subjects	None
3	T1D	Endocrine cells in T1D diabetes subjects	None
4	All-Small	Including only cells in islets with fewer cells than the median islet cell count found across all subjects	≤35
5	All-Big	Including only cells in islets with more cells than the median number calculated across all subjects	>35
6	Control-Small	Cells in islets from control subjects with fewer cells than the median number present in control subjects	≤34
7	Control-Big	Cells in islets from control subjects with more cells than the median number found in control subjects	>34
8	T1D-small	Cells in islets from T1D subjects with fewer than the median number found in all T1D subjects	≤37
9	T1D-big	Cells in islets from T1D subjects with more cells than the median number found in the T1D subjects	>37

ESM Table 10, in the first tab of the accompanying spreadsheet, contains the contingency tables for the nine group, as well as the p-values and odds ratio. The 95% confidence intervals of the odds ratio for the different group were calculated according to the formula [1]:

$$CI\pm = \exp\left[\log(OR) \pm 1.96\sqrt{\frac{1}{a}+\frac{1}{b}+\frac{1}{c}+\frac{1}{d}}\right]$$

Where OR is the odds ratio, the numbers a,b,c,d are those specified in ESM Table 5.

Quantification of the mantle core structure at islet level

Considering the same nominal variables as in the chi square test (cell type: beta/non-beta, cell position: mantle/core) but at islet level, a quantification of how much a given islet obeys to the mantle-core structure was proposed. The islet under study was fully characterised by the numbers defined in Table 3 (main text), namely, N, NB, M, NBm.

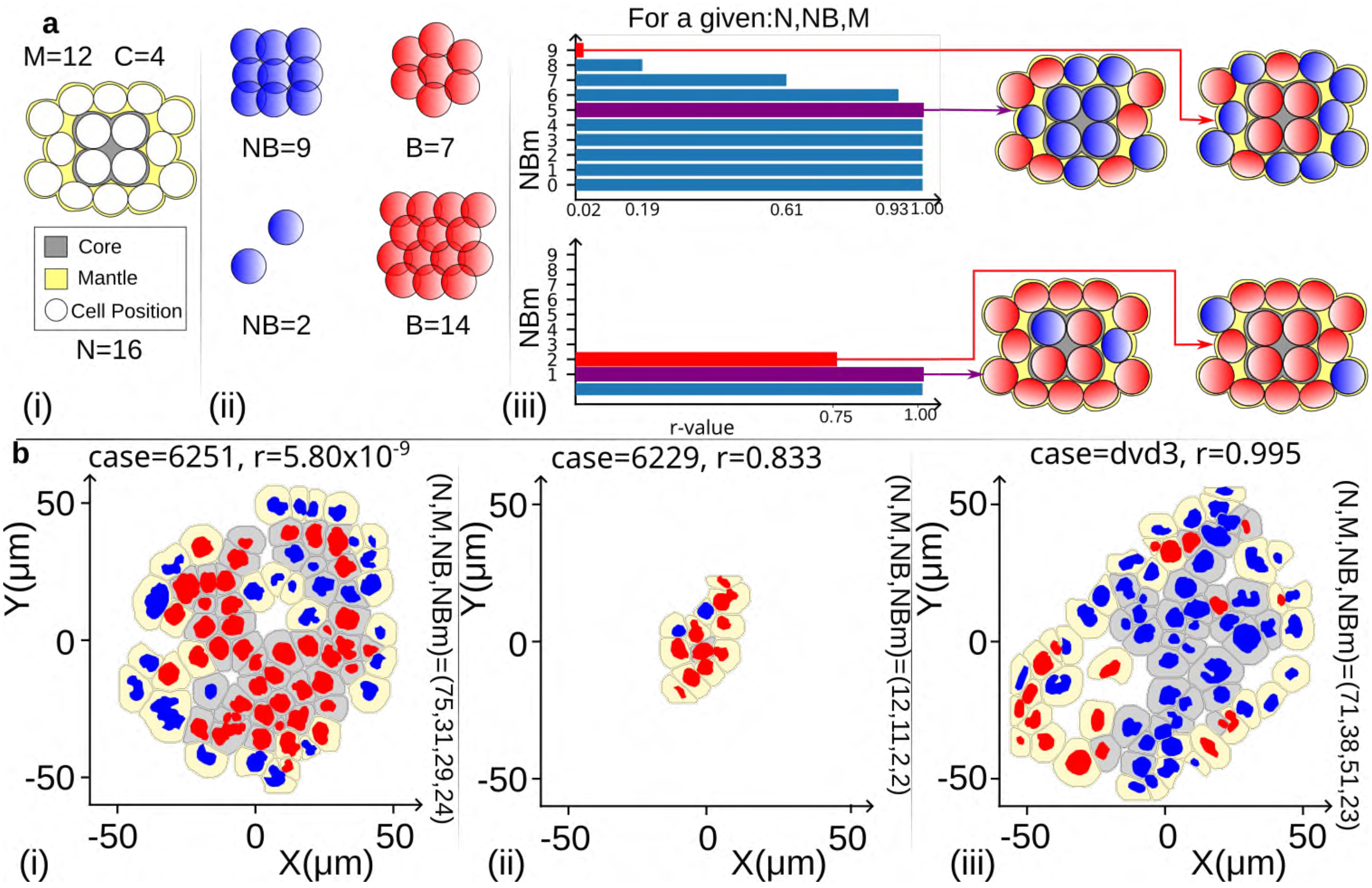

ESM Fig. 3: ***a*** *(i) Cartoon of the islet under study as a collection of positions (mantle: yellow, core: gray). Two possible populations of beta (red) and non-beta (blue) are proposed in (ii). (iii) Illustrates the probability of having at least k non beta cells at the mantle, computed for each islet combination. The rightmost panel shows possible arrangements corresponding to the respective probabilities, distinguished by coloured arrows.* ***b*** *Observed islet arrangements and their respective computation of the r values.*

To quantify the Mantle-core structure arrangement in an islet, it was instructive to picture the islet as a set of “spaces” of two types, namely, *core* and *mantle*, illustrated by gray and yellow spaces in Panel **a** of ESM Fig. 3). The cells, which are as many as the spaces, are considered separately belonging to the classes beta (red) and non-beta (blue). Hence, for a given set of spaces and cells, there are several possible arrangements (i.e. ways to place the cells in the spaces). When the cells are placed in the spaces randomly, the probability of having *exactly* **k** non-beta cells in the mantle, can be computed from the three numbers: N, M, and NB; using the hypergeometric distribution (For a general reference see any introductory text on probability and statistics such as [1]). The number is given by

$$P(k) = \frac{\begin{pmatrix} \mathrm{NB} \\ k \end{pmatrix} \begin{pmatrix} \mathrm{N} - \mathrm{NB} \\ \mathrm{M} - k \end{pmatrix}}{\begin{pmatrix} \mathrm{N} \\ \mathrm{M} \end{pmatrix}}$$

Using this distribution, it is possible to compute the probability of having *at least* **k** non beta cells in the mantle via the cumulative distribution, termed the r-value. ESM Table 11, in the second tab of the accompanying spreadsheet provides an implementation of the computation of the r-values, for the sample islets of ESM Fig. 3.

Unconstrained and digital siblings' islets

The computation of the r value depends on the number of cells (N), number of positions in the mantle (M), and number of non-beta cells (NB) in the islet. For certain combinations of these numbers, the r-value will always be one or very close to one. This is because the number of possible arrangements of the cells within the islet is highly *constrained*. These islets are excluded from the analysis. A trivial example of this occurs when the islet is composed only of beta cells. In this case the r-value, which computes the probability of having at least zero non-beta cells in the mantle, will always be one. Instead, if the islet owns only one non-beta cell, the probability of having at least one non-beta cell in the mantle is still considerably high. Similar scenarios occur when the islet is only (or almost only) composed of non-beta cells, or islets having almost only mantle positions (see inset (ii) in ESM Fig. 3 **b**).

Islets belonging to these categories are termed *constrained islet*, defined as islets with fewer than

- 3 beta cells.
- 3 non beta cells.
- 3 core positions.

Since they provide no information about the mantle-core structure, they are not considered in the analysis. Computing the r-value for the *unconstrained* islets provides a sample of r-values (see blue histogram in the bottom left of Panel **c**, Fig. 4, main text). Since the goal is to compare the distribution of the r-values against the case where the cells are arranged in a random manner, a population of “digital siblings” islets is generated to this end. For a given islet, characterised by the numbers N, M and NB, a random number of non beta cells in the border (NBm) is computed from the hypergeometric distribution. As a result, a similar sample of the r-values for the digital siblings is obtained (orange histogram in the bottom right of panel **c**, Fig. 4). The pronounced peak for small r-values is a good sign of the mantle-core structure. To compare the underlying distributions of r-values of the observed islets and digital siblings, the Earth Movers’ distance was employed.

Supplementary tables

ESM Table 7: *Summary of the ten cases considered in the study. In all cases, tissue was extracted from the pancreatic tail of Caucasian individuals. (ND: non-diabetic, T1D: type 1 diabetes). The rows corresponding to the control (type one diabetes) cases have been highlighted in light blue (red). Further details of the DiViD cohort are available in [2].*

No	Cohort	Case ID	Disease status	Gende r	T/Bank	Age@Dx	Age@Death	Cause of Death
1	NPOD	6048	ND	M	nPOD	NA	30	Cerebrovascular /Stroke
2	NPOD	6251	ND	F	nPOD	NA	33	Head trauma
3	NPOD	6229	ND	F	nPOD	NA	31	Head trauma
4	NPOD	6162	ND	M	nPOD	NA	22.7	Head trauma
5	DiViD (1)	BG11 2102	T1D	F	NA	25	Live Biopsy	x
6	DiViD (2)	BG12 06079	T1D	M	NA	24	Live Biopsy	x
7	DiViD (3)	BG12 04839	T1D	F	NA	34	Live Biopsy	x
8	DiViD (4)	BG12 05189	T1D	M	NA	31	Live Biopsy	x
9	DiViD (5)	BG12 05191	T1D	F	NA	24	Live Biopsy	x
10	DiViD (6)	Norge 1822	T1D	M	NA	35	Live Biopsy	x

ESM Table 8: *Reagents used in the study.*

ORDER of primary antibody application	HIER	Primary Antibody	Company (Cat#)	Primary Concent ration	Primary Incubation time (minutes at RT)	Fluoro-phore (OPAL)	Fluorophore Concentration (incubation 10mins)
First	Citrate pH6	Ghrelin	Biotechne (#MAB8200)	1/80	60	480	1/100
Second	Citrate pH6	Insulin	Thermo Fisher ICTABLS (#14-9769-82)	1/800	30	690	1/100
Third	Citrate pH6	CK19	(Abcam) Ab7754	1/500	60	620	1/100
Fourth	TE pH9	PP	Biotechne (#MAB62971)	1/100	60	570	1/80
Fifth	Citrate pH6	Glucagon	Abcam (#ab10988)	1/800	30	520	1/150
Sixth	TE pH9	SST	Santa cruz) (#Sc55565)	1/250	60	*780*	*1/80*
Nuclear Detection		Spectral DAPI	Akoya Opal Kit		10		*As per kit*

References

1. Walpole RE, Myers RH, Myers SL, Ye K (2007) Probability & Statistics for Engineers & Scientists, 9th ed. Pearson Education, Inc

2. Krogvold L, Edwin B, Buanes T, et al (2014) Pancreatic biopsy by minimal tail resection in live adult patients at the onset of type 1 diabetes: experiences from the DiViD study. Diabetologia 57(4):841–843. https://doi.org/10.1007/s00125-013-3155-y

Earth Mover's distance mini tutorial

The earth mover's distance (EMD), also called the Wasserstein distance, is a measure of dissimilarity between two frequency distributions. This distance can be estimated considering histograms obtained from samples of the two distributions of interest.

The purpose of this mini tutorial is to provide an intuitive introduction to the EMD to aid the understanding and interpretation of results (see Fig. 4 and 5) in the main text. The mathematical details have been purposely omitted. Three examples are considered building in complexity.

Example 1: EMD minimal example

Consider two samples of islets, obtained from two different populations. For each islet, the r-*value* is computed as described in the main text (i.e. r = the likelihood that an islet adheres to a M-C structure).

- Sample one contains 2 islets, with *r*-values: S1= {0.2, 0.8},
- Sample two contains 6 islets, with *r*-values: S2= {0.1, 0.15, 0.9, 0.95, 0.3, 0.6}.

The r-values are binned into 4 equidistant bins, specified in ESM Table 9.

ESM Table 9: *binning of the r-values and respective indices.*

r-value	index
$0 \leq r \leq 0.25$	1
$0.25 < r \leq 0.5$	2
$0.5 < r \leq 0.75$	3
$0.75 < r \leq 1$	4

The histogram of each sample is represented in ESM Fig. 4 **a**. The goal is to measure the dissimilarity between the distribution of samples S1 and S2 via EMD. A necessary first step is to make sure that the area covered by both histograms (in red) is the same.

In this example, these areas are different due to the different number of islets in each sample, and so a rescaling of the histograms is required. In each histogram of panel **a**, the left and right vertical axis correspond to the number of islets in each bin for the sample, and the fraction of islets in the sample, respectively. The EMD looks to answer the question of what the minimal redistribution of the red area needs to be carried out to turn one of the histograms into the other one. Although the EMD is symmetric (i.e. the total amount of earth to transport from the histogram of S1 into the one of S2, is the same as the amount needed to transform S2 into S1), usually the histogram to be redistributed (where the earth will be moved) is referred as the *source,* and the histogram sought to replicate using the redistribution is the *target* histogram.

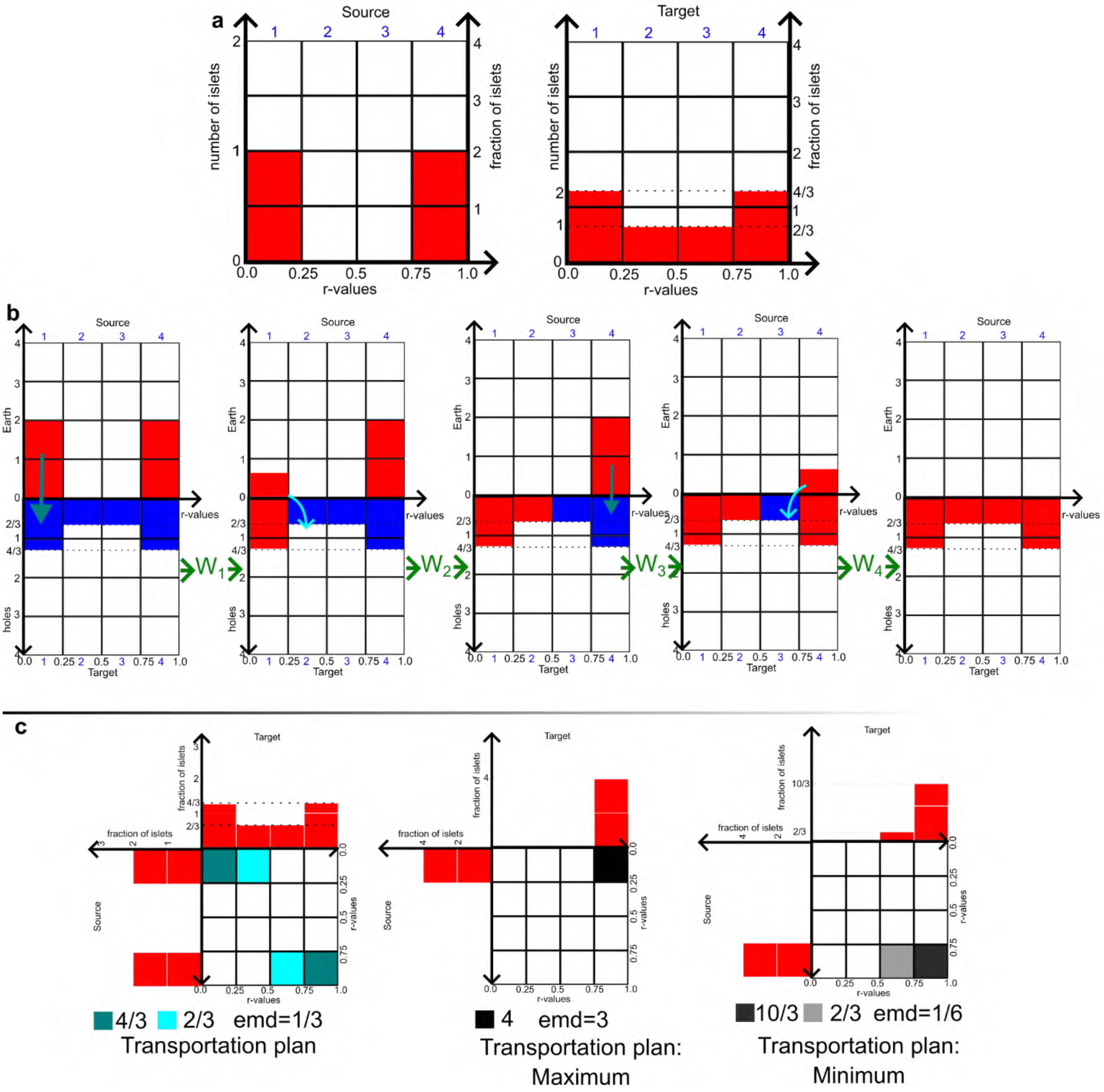

***ESM Fig. 4,** Example 1: **a** Source (left) and target (right) histograms to compare via EMD. The y axes on the left and right show the total number and fraction of islets, respectively. **b** Step by step transportation plan. The arrows, coloured according to the total mass transported (see left plot in **c**), illustrate the transport of earth from the source histogram (on top on red) to the target (blue holes, bottom) to move to the next step. **c** Matrix representation of the transportation plan. The leftmost panel illustrates the case in **a**. Middle and right panels show two configurations of this example, leading to the maximum (middle) and minimum non-zero (rightmost) EMDs.*

At this point, it is useful to use the analogy of the earth mover's distance. Imagine the source histogram (left histogram in **a**) as piles of earth (i.e. the first and fourth bin have 2 units of earth, bins 2 and 3 have zero). To begin the transportation of the earth, the target (right) histogram is transformed into holes beneath the piles of earth, represented in blue relief (leftmost inset in panel **b**). The goal is to create a plan to cover all the holes (blue) using the earth (red), while minimizing the *work* ***W****, defined as:*

$$\boldsymbol{W} = \text{mass x displacement},$$

where the mass (also referred as earth) is the fraction of the histogram transported, and the displacement is the distance between the donor and recipient bins (chosen to be the distance

between the centers of the bins in this example). As it should be clear from the representation in panel **b**, the transportation plan that minimises the work corresponds to:

- **W_1:** Use 4/3 of the 2 units of earth in bin 1 to cover the hole in bin 1 (see first and second inset from the left in panel **b**). Since the displacement between bin one to bin one is zero, the work for this action is zero. **W_1**=0.
- **W_2:** Transport the reminding 2/3 units of earth in bin 1 to bin 2 (see second and third panels in **b**). Since the distance between bin 1 and 2 is 0.25, **W_2**= 2/3*1/4.
- **W_3**: Use 4/3 of the 2 units of earth in bin 4 to cover the hole in bin 4. Similarly to **W_1**, here **W_3**=0.
- **W_4:** Transport the reminding 2/3 units of earth in bin 4 to bin 3. Similar to **W_2**, here **W_4**=2/3*1/4.

The total EMD corresponds to the total work, this is:

$$EMD=\mathbf{W_1}+\mathbf{W_2}+\mathbf{W_3}+\mathbf{W_4}=1/3$$

This transportation plan can be represented using a matrix. The leftmost plot of panel **c** in ESM Fig. 4 illustrates this. On the left and top of the matrix, the source and target histograms have been depicted. The values of the matrix show how much and where earth must be redistributed from the *source* to the *target*. For two identical histograms, the transport plan is a diagonal matrix. In this example, the first row shows that 4/3 of earth should stay in bin 1 and 2/3 should be transported to bin 2. The last row shows that 2/3 must be transported from bin 4 to bins 3, and 4/3 must remain in bin 4.

Next, consider which 2 samples would lead to the maximum and minimum non-zero EMD, in this setup?

The maximum distance corresponds to the case where all the earth in the source is as far away as possible from the earth in the target. In this case, the transportation plan (middle plot in panel **c**) corresponds to moving all the earth as far as possible (3 bins in this case). Consequently, the EMD=4*3/4 =4.

Strictly speaking, there is no non-zero minimum distance when considering two different distributions. However, since in this example sample 1and 2 have 2 and 6 islets, in practice, the closest two histograms can be without being identical corresponds to the case where they differ in only one islet. For instance, when the target histogram has a single islet "one bin away" from the source distribution. An example of such configuration has been included in the rightmost plot of panel **c**. In such a case, the EMD corresponds to the minimal possible work admitted by the grid. This is the minimal amount of earth (a single islet in our case) times the minimal displacement (distance between two neighbour bins), corresponding to EMD=1/6 in this example.

Example 2: EMD on large samples

The first example introduced key concepts in the computation of the EMD such as the bins, earth (mass), work, and the transportation plan. In all the cases considered, the transportation plan was straightforward to compute intuitively.

As the sample size increases, finding the transportation plan which minimises the work becomes a challenge. Thankfully, this problem can be solved via optimisation algorithms already

implemented in several packages. In this mini tutorial, the Python Optimal Transport (POT) library is considered. Any alternative package must provide identical results.

Consider 2 samples of r-values obtained for two populations of islets. The first sample S1 contains 300 observations, and the second sample S2 contains 100. Assume that somehow it is known that the distribution of r-values in each case obeys to a normal distribution, characterised by the mean and standard deviations (std):

S1: mean=0.3 std=0.12   *S2: mean=0.8, std=0.1*

In what follows, we consider equally spaced bins. As in the first example, the total area under the curve for both distributions must be the same. Instead of forcing the total area occupied by the rectangles to be one, in this example, the total number of islets in each bar is divided by the total number of islets in the sample. In this way, the total amount of earth transported never exceeds unity. This is the normalisation used in the main text. ESM Fig. 5 **a** shows the histograms of S1 and S2 in terms of number of islets (left y-axis) and proportion of islets (right y-axis).

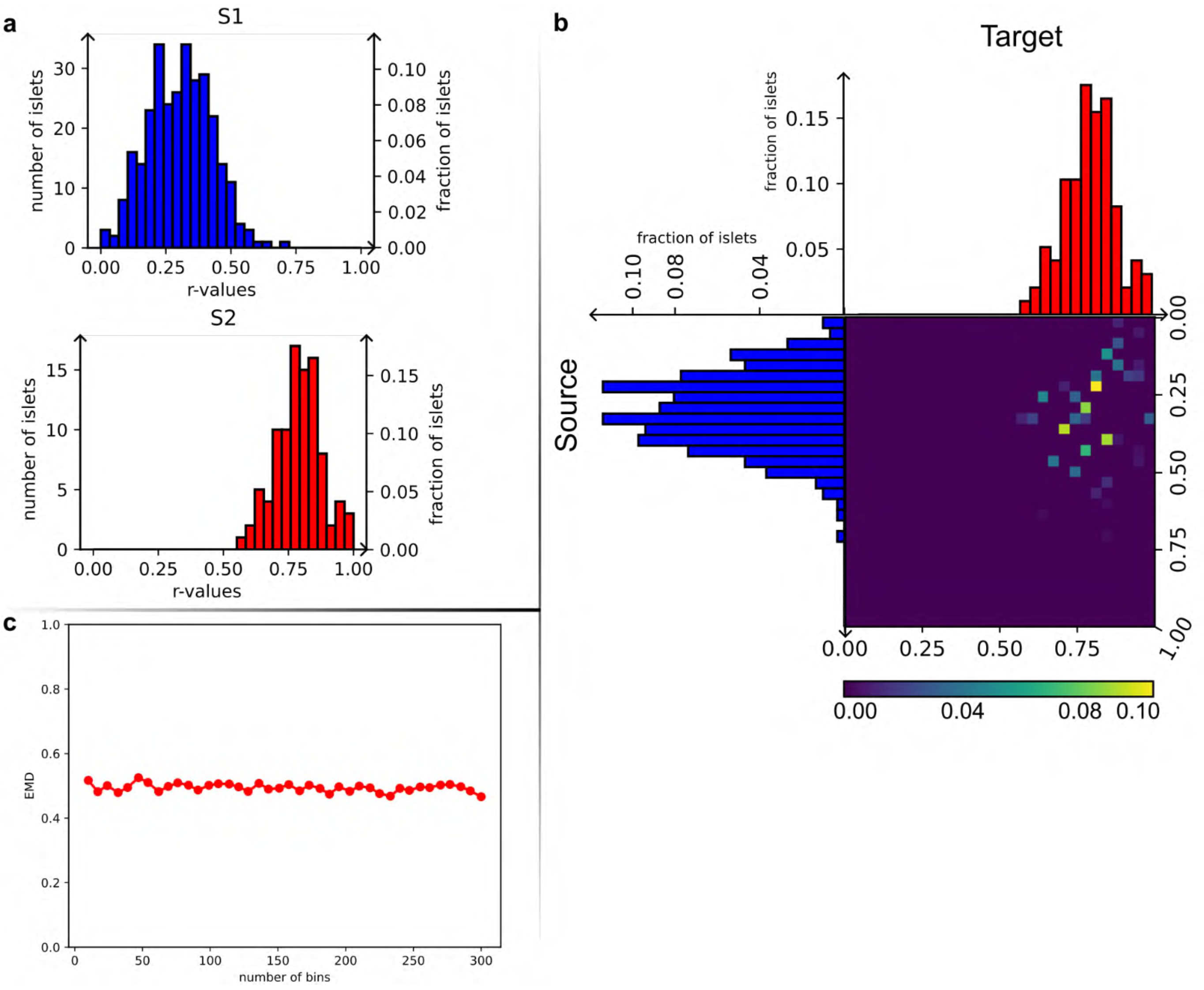

ESM Fig. 5: *Computation of the EMD, considering large (in the orders of hundreds of islets) samples of r-values.* ***a*** *Histogram of the total number of islets for samples S1 (top in blue) and S2 (bottom in red), counting the total number of islet (left vertical axis) and fraction of the total number of islets (right vertical axis) in the sample.* ***b*** *illustration of the transportation plan, computed using EMD. The earth movers' distance in this example is roughly 0.49.* ***c*** *Computation of EMD for different number of bins.*

Once the histograms have been computed, the algorithm needs a matrix accounting for the distances between bins. Considering the histograms and matrix of distances, the optimisation plan and EMD can be computed. An example of the transportation plan is illustrated in panel **b** of ESM Fig. 5. A natural question is how dependent the EMD on the sample size is. Panel **c** of ESM Fig. 5 shows that the number of bins does not significantly affect the EMD. This was also illustrated in the results in the main text.

Example 3: Minimal example with histograms of two variables

For the last example, two samples of islet are considered. The first sample S1 contains two islets and the second sample S2 only one. For each islet in the experiment, two quantities are measured, namely, the r-value and the fraction of beta cells in the islet. The samples are

S1={(0.3,0.4), (0.9,0.8)};   S2={(0.3,0.7)},

where the first number is the r-value and the second is the fraction of beta cells. Considering 4 equispaced bins in both directions, the histograms of the Source (S1) and target (S2) samples are presented in panels **a** and **b** of ESM Fig. 6 (the total number of islets has been rescaled such that the sum of all squares is one). In contrast with the single variable examples above, here transportations are possible along the horizontal and vertical directions. Consequently, the maximum distance corresponds to the maximum distance in the vertical direction, plus the maximum distance in the horizontal one. Since the total mass is rescale to one, the maximum EMD is always less than the maximum distance, and consequently 0≤EMD(2D) ≤2.

Similarly to the first example, here the optimal plan is trivial:

1. **W_1:** Move the fraction of islets from the bin (4,4) into the position of the black bin, located at (2,3), in the target. This movement involves transporting mass across two bins on the horizontal direction and one bin on the vertical. Hence for a mass of 1/2, the work is: **W_1**=1/2 x (2/4+1/4)=3/8.
2. **W_2:** Move the islets from the square (2,2) into the black square of target (2,3). This movement requires only one move vertically. Hence, for a mass of 1/2, the work is **W_2**=1/2 *1/4=1/8.

The EMD is given by the total work EMD=**W_1**+**W_2**=1/2.

The problem of computing EMD for samples of two variables is analogous to a single variable. The main difficulty lies in the visualisation of the results. Because a heatmap is needed to represent the histogram with two variables, it is not possible to have a visualisation of the transportation plan in the same way as for the single-variable case (e.g. ESM Fig. 5 **b**). In simple cases (like the one consider here), it is possible to illustrate the transport with arrows (e.g. Panel **c**). In general, this is not *possible.* A common strategy is to show the parts of the transportation plan requiring most of the total work, as these should reflect the main differences between distributions. In two dimensions, the EMD is not significantly affected by the number of bins nor the size of the sample. The maximum and minimum EMD in 2D are analogous to the 1D case (shown in Example 1). For instance, the maximum distance between 2 samples in the current example corresponds to a source with only islets in the bin (1,1) and target with all islets in bin (4,4). The work needed to move the total mass (1) from (1,1) to (4,4) is all the bins on the horizontal, plus all the bins in the vertical times the mass

Maximum EMD= (3/4+3/4)x1=1.5

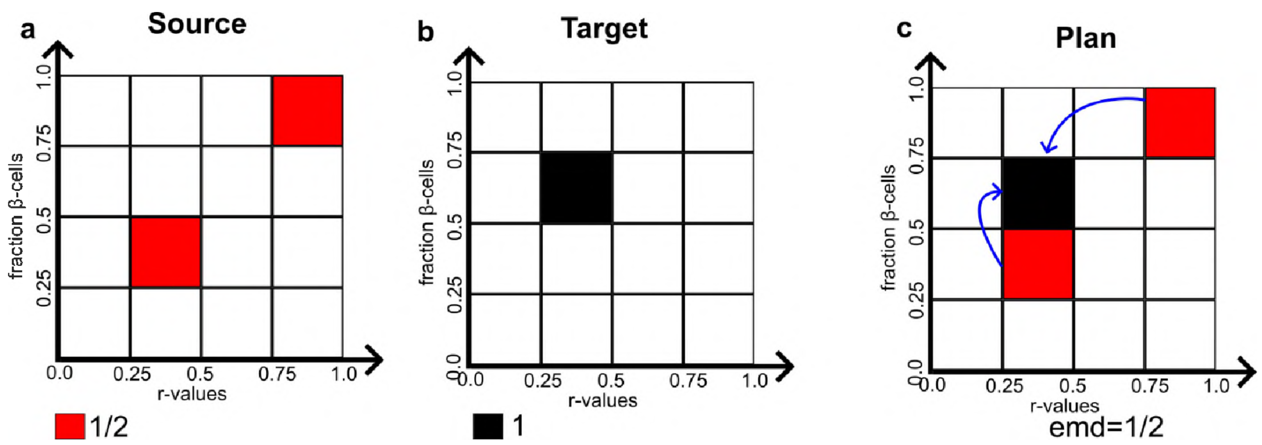

ESM Fig. 6: Computation of EMD considering simple histograms with two variables (heatmaps). Source (red) and target histograms are illustrated in panels **a** and **b**, and the transportation plan in **c**.
